# The Genetic Factors of the Airway Epithelium Associated with the Pathology of Asthma

**DOI:** 10.3390/genes13101870

**Published:** 2022-10-15

**Authors:** Maral Ranjbar, Christiane E. Whetstone, Hafsa Omer, Lucy Power, Ruth P. Cusack, Gail M. Gauvreau

**Affiliations:** 1 Department of Medicine, McMaster University, Hamilton, ON L8N 3Z5, Canada; 2 Respirology Department, Galway University Hospital, H91 YR71 Galway, Ireland

**Keywords:** asthma, genetics, single nucleotide polymorphisms (SNPs), association studies, treatment response, gene-environment interaction

## Abstract

Asthma is a chronic disease of the airways characterized by inflammation, tightened muscles, and thickened airway walls leading to symptoms such as shortness of breath, chest tightness, and cough in patients. The increased risk of asthma in children of asthmatics parents supports the existence of genetic factors involved in the pathogenesis of this disease. Genome-wide association studies have discovered several single nucleotide polymorphisms associated with asthma. These polymorphisms occur within several genes and can contribute to different asthma phenotypes, affect disease severity, and clinical response to different therapies. The complexity in the etiology of asthma also results from interactions between environmental and genetic factors. Environmental exposures have been shown to increase the prevalence of asthma in individuals who are genetically susceptible. This review summarizes what is currently known about the genetics of asthma in relation to risk, response to common treatments, and gene-environmental interactions.

## 1. Introduction

Asthma is a heterogenous inflammatory disease of the airways characterized by airways hyperresponsiveness, reversible airflow obstruction, and mucus hypersecretion [1]. Poorly controlled asthma may be adversely impacted by several comorbidities including rhinosinusitis, gastro-oesophageal reflux disease (GERD), obstructive sleep apnea (OSA), psychological dysfunction, and chronic infections [2]. Asthma has affected approximately 262 million individuals in 2019 and its prevalence has been increasing worldwide [3]. The incidence of asthma is significantly higher among children and adolescents as these populations are highly exposed to allergens, including air pollution and tobacco smoke, and their immune system is not trained to function properly when triggered by such allergens [3]. It has long been known that asthma is a complex disease with several risk factors involved in its etiology. It was first confirmed by a study in twins that asthma has a hereditary component. In other words, genetic predispositions contribute to the development of asthma and this heritability rates in the adult population of up to 60 percent [4].

The airway epithelial cells are the first barrier against pathogens and environmental insults. Any damage to the epithelium promotes the release of alarmin cytokines, including TSLP, IL-33, and IL-25. These cytokines activate a variety of immune cells and downstream inflammatory pathways. Activated Mast cells and basophils release mediators, including histamine, prostaglandin (PG) D_2_ and leukotrienes, which contract airway smooth muscle and increase mucus secretion. Drugs targeting these mediators, including SABA, LABA, and anti-leukotrienes, prevent the development of these pathways [5].

**Figure 1:**
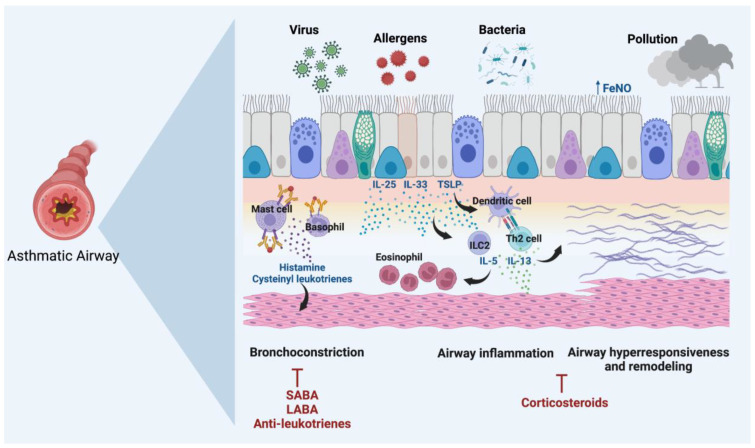
Pathophysiology of asthma. Alarmin cytokines IL-25, IL-33 and TSLP are released from the airway epithelium in response to external stimuli such as pathogens, allergens, and pollution. These cytokines result in the downstream production of type 2 cytokines (IL-4, IL-5, and IL-13) from Th2 cells and their innate counterpart, ILC2s. The type 2 cytokines induce airway inflammation, FeNO, airway hyperresponsiveness, and remodeling, all inhibited by corticosteroids. The production of histamine and leukotrienes from activated basophils and mast cells promotes bronchoconstriction, which is prevented by antagonists. FeNO = fractional exhaled nitric oxide; LABA = long acting β-agonist; SABA = short-acting β-agonis.

Alarmin cytokines also promote the activation of group 2 innate lymphoid cells (ILC2s), the maturation of CD4^+^ T cells into Th2 cells, and the production of Type 2 cytokines (IL-4, IL-5, and IL-13). Type 2 cytokines result in airway eosinophilia, mucus overproduction, bronchial hyperresponsiveness (BHR), and remodeling of the epithelium and subepithelial matrix, which can be suppressed by glucocorticoids [6] (Figure 1).

The presence of such genetic factors associated with asthma implies that understanding the genetic basis of this disease might explain many mechanisms affecting the pathology of this disease. In the past decades, there has been an increased interest to obtain a better understanding of the genetic risk factors involved in asthma. The majority of these risk factors are single variations occurring within the DNA, which are known as single-nucleotide polymorphisms [SNPs], and these genetic variants have an allele frequency of more than 1% in the population [7]. With the help of next-generation sequencing technology in recent years, genome-wide association studies (GWASs) have become a novel approach to identify associations between genotypes and phenotypes [8]. The primary methods for studying complex disease-susceptibility genes include GWASs, candidate gene association studies, and genome-wide linkage analyses, which can provide insight into disease-causing mechanisms and, eventually, novel targets for asthma therapeutics. 

In this review paper, we summarize the data on the genetic single nucleotide polymorphisms responsible for the susceptibility to asthma, the patients’ responses to treatments in the presence of different genetic factors, and gene-environment interactions involved in the pathobiology of asthma. 

## 2. Genetic Polymorphisms Associated with Asthma Susceptibility and Severity 

There are several genes that regulate asthma, and others that contribute to disease risk. Moreover, clinical findings have shown that asthma severity varies among individuals and may also change over time. Among the susceptibility genes, there are those that are known to significantly increase the risk of asthma. Genetic polymorphisms in genes including alarmin cytokines (TSLP and IL-33) type 2 cytokines (IL-4, IL-13) and other inflammatory-related proteins (HLA, ADAM33), and vitamin D receptor have been shown to enhance, or reduce, the risk and severity of asthma in individuals.

### 2.1. Thymic Stromal Lymphopoietin (TSLP)

Thymic stromal lymphopoietin (TSLP) is an epithelial-derived cytokine that is produced following exposure to external stimuli such as bacteria, viruses, smoke, and allergens [9]. TSLP drives allergic inflammation through binding to the TSLP receptor on inflammatory cells, including mast cells, dendritic cells, and eosinophils. TSLP expression is increased in patients with asthma compared to healthy controls and correlates with measures of the airway obstruction [10]. An anti-human TSLP monoclonal antibody (AMG157/tezepelumab) that prevents the interaction of TSLP with its receptor was shown to significantly reduce allergen-induced airway responses and airway inflammation in patients with mild allergic asthma [11]. Tezepelumab has been approved for use in severe asthma following clinical trials showing a reduction in asthma exacerbation, improvement in lung function and asthma control, and reduction of blood eosinophils and airway fractionated nitric oxide (FeNO) levels [12].

Genetic studies have shown that TSLP gene variants play a role in the development of asthma and are associated with the risk and severity of this disease, however, the results are inconsistent. A study published by Harada et al. demonstrated that two single nucleotide polymorphisms existing at the promoter region of the TSLP gene (rs3806933 and rs2289276) were positively associated with asthma susceptibility in children and adult asthmatics [13], this positive or direct association can be explained as a higher risk of asthma in individuals carrying these SNPs. The results of this study were expanded by Birben et al. showing that the association with these SNPs is sex-specific with the CC genotype of SNP rs3806933 positively associated with asthma in the male population, while the CC genotype of rs2289276 correlated with higher eosinophil counts in female subjects [14]. While these two studies showed a positive association between the TSLP polymorphisms and asthma, a study conducted by Hunninghake et al. and others contradicted the previous findings by showing that two SNPs in the genomic region of TSLP (rs1837253 and rs2289276) were inversely correlated with asthma and thereby making them less susceptible to developing asthma [15,16,17]. Moorehead et al. investigated the expression of the SNP rs1837253 in the TSLP gene, and its association with asthma, allergic disease, and eosinophilia [16]. The authors found protective effects in individuals carrying rs1837253, and speculated this may be related to the changes happening in the production of the long isoform of TSLP, which ultimately leads to reduced production of this protein [16]. 

Overall, more studies are required to discover the functional effects of each polymorphism, which could provide a basis for investigating the clinical effects of anti-TSLP therapies in different TSLP gene variant groups.

### 2.2. Interleukin-33 (IL-33)

IL-33 is an epithelial-derived alarmin cytokine that plays a significant role in the pathobiology of allergic disease and asthma. IL-33 is secreted by epithelial cells and inflammatory cells in the airways and plays a role in type-2 innate immunity by activation of mast cells, group 2 innate lymphoid cells [ILC2], basophils, eosinophils, and macrophages through the ST2 receptor [18]. IL-33 is one of the earliest cytokines released in response to stimulation with allergens [19], with elevated levels found in the airway secretions of patients with acute asthma, and these elevated levels of IL-33 expression in lung epithelium correlates with asthma severity [20].

GWAS have shown that polymorphisms in the IL-33 gene are associated with asthma susceptibility [21], however the impacts of these polymorphisms are not restricted to the risk of asthma. A study conducted by Wu et al. [22] demonstrated that rs4742170 was associated with higher FENO levels, and a worse response to ICS treatments, indicating that SNPs may also affect the severity and treatment response in patients. A larger study conducted in Finland, including 521 cases of adult onset asthma and 1016 controls reported a direct association between SNPs rs996029, rs2006682, rs7037276, and adult onset asthma [23]. The same correlation has been demonstrated for the SNPs rs3939286 and rs1342326 [24]. 

In contrast to SNPs evaluated in the previous studies, a whole genome sequencing project in Iceland found the IL-33 SNP rs146597587 was associated with lower eosinophil counts in children, suggesting expression of this SNP provides protection from developing asthma [25]. The same inverse association between IL-33 SNPs and asthma was reported by Queiroz et al. [26]. The protective or indirect association would suggest a lower risk of developing asthma in those who have these SNPs present in their genome. 

### 2.3. Interleukin 1 Receptor-like 1 (IL1RL1)

IL1RL1, also known as ST2, is a member of the IL-1 family and serves as the receptor for the epithelial-derived IL-33 cytokine. There are 3 different subtypes of ST2 expressed in the lungs; sST2, IL1RL1-b, and IL1RL1-c. sST2 is a soluble form and acts as a decoy receptor preventing the IL-33 from binding to the receptors expressed on immune cells. IL1RL1-b is the main receptor expressed on the surface of immune cells. The binding of IL-33 to IL1RL1-b will activate the MyD88/NFkB pathway, resulting in the downstream activation of cells involved in type2 immune response, thus promoting airway inflammation [27]. 

In a Chinese population, Zhang et al. reported a significant positive association between two SNPs in the IL1RL1 gene, rs1420102, and rs13431828, with an increased risk of asthma [28]. Another study conducted in a Chinese Han population reported that two SNPs located in the intron of IL1RL1 (rs10208293 and rs13424006) were associated with eosinophilic inflammation and childhood asthma [22]. The functional mechanism of these two SNPs is not fully understood; however, it can be hypothesized that these variants might play a role in regulating the production of the sST2 receptor, which serves as a decoy receptor to reduce free IL-33. 

A study performed on the European population by Savenije et al. had shown a positive correlation between two SNPs [rs10513854 and rs9290936] and persistent wheeze in childhood asthma [17]. However, these findings could not be replicated in Wu et al.’s study in a Chinese Han population, and this discrepancy may be related to the small sample size or ethnic group studied. Since the prevalence of mutant genotypes of SNPs is normally low in populations, a small number of subjects may not be sufficient to show any significant differences between patients and the healthy control group. This issue could also explain the lack of any significant associations between IL1RL1 gene variants and asthma in a study of a Puerto Rican population [29]. 

Moreover, there are different variants in the IL1RL1 gene that have demonstrated protective associations; in a European study reported by Savenije et al., rs11685480 and rs1420102 correlated with lower counts of blood eosinophils, indicating a decreased risk of developing severe asthma. They also showed that these variants impact the serum level of sST2 [30]. The effect of IL1RL1 gene polymorphisms on serum levels has also been reported by Dijk et al. which showed significant associations between rs13431828, rs1420101, rs1921622, and rs10204137 and lower serum sST2 levels. It can be concluded that gene variants can exert their effects by regulating the protein expression [31]. Dijk has also reported that the SNP rs13431828 can promote a more severe phenotype of asthma by increasing the risk of exacerbations and hospitalizations in children [32].

### 2.4. Interleukin-13 (IL-13)

Interleukin-13 is an inflammatory cytokine produced by type 2 T helper cells and group 2 innate lymphoid cells. The IL-13 gene is located on chromosome 5 and contains four exons and three introns. IL-13 shares similar secondary structures with IL-4, which is also an important cytokine in allergic inflammation, and both IL-13 and IL-4 exert their effects through a shared receptor. The binding of IL-13 to its receptor in the airway epithelium of asthmatic patients leads to mucus hypersecretion and promotion of bronchial hyperresponsiveness [33]. 

A genome-wide association study has highlighted the essential role of the SNP rs1295686, located in the intron of the IL-13 gene, in the impairment of total IgE production [34]. The finding of this GWAS study prompted further genetic studies to replicate the results in different ethnic populations, which confirmed and extended the findings of the GWAS study to show an increased risk of asthma among children and adults carrying the SNP rs1295686 in Asian, Caucasian, and middle eastern ethnicities [35,36,37,38]. Similarly, rs20541 is significantly associated with a higher risk of asthma and airway hyperresponsiveness [35,39]. It is suggested that the single change in the nucleotide sequence of the IL-13 gene substitutes the arginine amino acid with glutamine, increasing the receptor binding affinity of the secreted cytokine which ultimately increases the downstream inflammatory activity [40] Imraish et al., however, report a significant reduction in IgE serum levels in asthmatics carrying the minor allele of the rs20541, suggesting an inverse relationship between this SNP and the severity of asthma [41]. The mechanisms by which this SNP affects asthma risk are not clear yet.

### 2.5. Interleukin-4 (IL-4)

Interleukin-4 (IL-4) is a pivotal cytokine in allergic inflammation with a central role in driving the differentiation of naïve T helper cells into Type 2 helper T cells and in the isotype switching of B cells to IgE antibody production. Production of IL-4 in the asthmatic airway epithelium results in production of chemokines from epithelial cells, increased airway eosinophil numbers, and increased bronchial hyperresponsiveness [42]. The IL-4 gene is located on chromosome 5 in a region that has been reported to be correlated with asthma and other allergic disorders [43]. 

Among the different single nucleotide polymorphisms discovered in the IL-4 gene, rs2070874, located on the UTR region of the gene, has been shown to be associated with higher serum IgE levels and increased risk of asthma in different ethnic populations [44,45]. Another SNP in the promoter region of the IL-4 gene (rs2243250) has been shown to increase the risk of asthma in children and adults [46,47]. This direct pattern has been replicated by Michael et al. also reporting a direct association for allergic rhinitis in a population of 106 patients with allergic rhinitis [48]. The mechanism by which this polymorphism increases the asthma risk has been explained by Rosenwasser et al. demonstrating an increase in the binding of transcription factors to the promoter region when this SNP is present. It can be concluded that increased transcription factor binding will lead to increased production of IL-4 cytokine and, therefore, upregulation of inflammatory responses [46]. 

In contrast, the rs2227284 SNP in the IL-4 gene was reported to have an inverse and protective association with asthma by decreasing the risk of asthma in carriers, and furthermore, the protective effect of rs2227284 was more pronounced in carriers with homozygous mutant genotype compared to a heterozygous genotype [47].

### 2.6. The Human Leukocyte Antigen (HLA)

The airway epithelium is exposed to a large number of inhaled antigens. Human leukocyte antigens (HLA) are a genomic region located on chromosome 6, encoding a large variety of proteins important in the regulation of the immune system. In general, the main role of HLA molecules is to present antigens to CD4^+^ and CD8^+^ T lymphocytes, where HLA class I molecules present peptides originating from inside the cells to CD8^+^ cytotoxic T cells and HLA class II molecules present the antigens that have entered the cells from outside to CD4^+^ T helper lymphocytes [49]. 

HLA loci are considered highly polymorphic regions that are linked to susceptibility to immunological disorders, transplantation success, host defense, and atopic diseases such as asthma. Several studies have shown that various HLA alleles are associated with asthma [50]. A large GWAS has identified single nucleotide polymorphisms in the HLA-DQ locus that are highly associated with an increased risk of asthma. SNP rs9272346, located near the HLA-DQA1 locus, is one of the SNPs shown to have a significant association with both adult and childhood asthma [51]. HLA-DQA1 locus is among the MHC class II loci with the most polymorphisms linked to asthma and allergic disease. Several studies have demonstrated a positive correlation between HLA-DQA1* 0101, DQA1 * 0601, HLA-DQB1 * 0303, DQB1 * 0601, and HLA-DQB1 * 0201 alleles and susceptibility to asthma in diverse ethnic populations [52,53,54,55]. 

### 2.7. A Disintegrin and Metalloproteinase 33 (ADAM33)

The ADAM33 gene located on chromosome 20 was one of the first genes identified to be associated with asthma. ADAM33 plays several important roles in cell activation, proteolysis, adhesion, fusion, and signaling. It has been shown that the expression of ADAM33 is increased in the epithelium and airway smooth muscles of asthmatics, suggesting a role in the pathogenesis of bronchial hyperresponsiveness and airway remodeling [56]. Studies have shown that ADAM33 levels are higher in asthmatic patients and correlate with serum levels of IL-4 and IL-13, suggesting that its expression is regulated by Th2 cytokines [57]. Taken together, ADAM33 is considered an important element in the promotion of asthmatic response, and therefore, genetic variations throughout its gene may be linked to asthma susceptibility and severity. 

A genome-wide evaluation of 460 Caucasian families by identified several polymorphisms within the ADAM33 gene associated with asthma susceptibility [58]. Following the findings of this study, many candidate gene studies were conducted in different populations to replicate the results; however, there were several inconsistent reports, potentially resulting from ethnic differences and smaller sample sizes. There was no association between any of the four ADAM33 SNPs and asthma in the Iranian population [59]. Similarly, a German study did not find any correlations between SNPs of the ADAM33 gene and childhood asthma, despite a large study population [60]. Shen B et al., reported a significant association between SNP rs44707 and severe asthma, as well as rs2787094 with less severe asthma, in an Asian population [61], and similar results were reported in a Thai study in which rs44707 was associated with the high severity group [62]. Furthermore, there was a significant correlation between F+2, rs44707, and V4 variants and the risk of asthma in the Indian population [63]. The same significant association for rs44707 was equally reported by Fedorova et al., who also reported the prevalence of haplotypes rs44707, rs2787095, rs2485700, and rs2280091 was higher in the healthy control compared to the asthmatics suggesting a protective effect against asthma in those carrying this haplotype [64]. In a study consisting of 96 asthmatics and 86 healthy children, Ning et al. reported a significant correlation between SNP rs678881 and increased risk of childhood asthma but no significant differences in the prevalence of rs2280089 and rs2853209 between the two groups [65]. 

### 2.8. Vitamin D Receptor (VDR)

Vitamin D is a steroid soluble nutrient predominantly located in the skin with the function of calcium absorption and bone health. It is also reported to have immunomodulatory effects on both innate and adaptive immunity. Vitamin D metabolism in airway epithelium is shown to increase the lung immune response and therefore modulate the production of inflammatory cytokines from these cells [66]. A Cochrane review of vitamin D in asthma found that while there was no effect on lung function or asthma control, oral vitamin D supplementation reduced the risk of severe asthma exacerbations requiring systemic corticosteroids and hospital admission, with no adverse effects reported, suggesting it is a safe and potentially effective intervention [67]. 

The vitamin D receptor [VDR] gene is located in chromosome 12 near asthma-related genes suggesting that polymorphisms in this gene may be a good indicator of asthma occurrence. GWAS has identified four single nucleotide polymorphisms within the VDR gene that are shown to be related to asthma risk and severity. These SNPs include TaqI [rs731236], ApaI (rs7975232), FokI (rs2228570) and BsmI (rs1544410) [68]. Findings from a study conducted in Turkey provide evidence for an association between *Taq*I and *Apa*I polymorphisms and asthma susceptibility. These polymorphisms were also shown to be correlated with lower mRNA gene expression of VDR [69]. The significant association between TaqI and asthma susceptibility was also reported by other studies [70,71,72]. 

Pillai et al., reported that VDR polymorphisms may affect the severity of asthma, demonstrating that rs7975232, rs2239185, rs2107301, rs1540339, rs3782905, and rs2228570 were significantly associated with lower pre-bronchodilator spirometry and greater reversibility, both indicators of increased risk of asthma severity [73]. Taken together, the polymorphisms within the vitamin D receptor gene play a role in the susceptibility to asthma, and lead to an increased risk of asthma in the carriers. 

**Figure 2:**
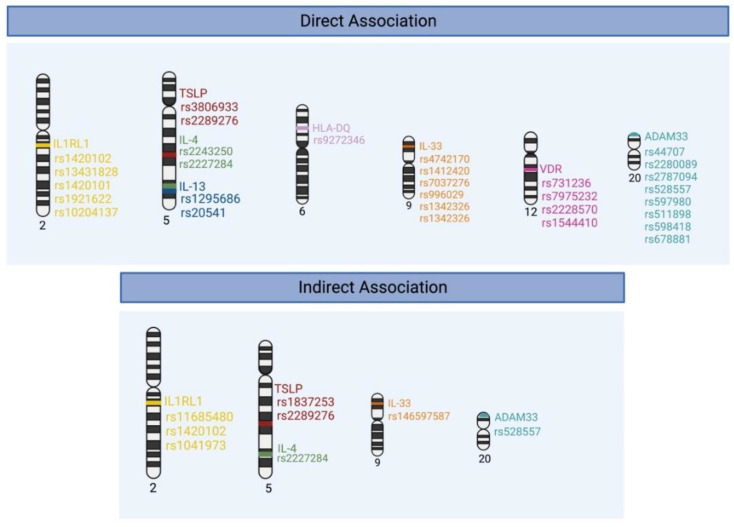
Summary of SNPs associated with asthma. Some SNPs have been reported to have both direct and indirect effects based on the study.

A summary of the genetic polymorphisms in the alarmin cytokines (TSLP and IL-33), type 2 cytokines (IL-4, IL-13) as well as HLA, ADAM33, and vitamin D receptor are summarized (Table 1, Figure 2). 

## 3. Genetic Polymorphisms Associated with Asthma Treatment Response 

Treatment for asthma is aimed at either bronchoconstriction or airway inflammation components of the disease. Spasmogens such as histamine, cysteinyl leukotrienes, and prostaglandin D2 released from airway mast cells and basophils promote smooth muscle contraction resulting in bronchoconstriction [78], which can be prevented by treatment with β-2 agonists and anti-leukotrienes. Activation of the epithelium and resident inflammatory cells leads to the accumulation of immune cells, including eosinophils, lymphocytes, macrophages, neutrophils, and basophils, which support an inflamed tissue environment and contribute to mucus hypersecretion, airway hyperresponsiveness, and remodeling. Corticosteroids are effective for suppressing airway inflammation and thereby preventing asthma exacerbations [79]. The variable rate of response to asthma therapies across the patient population can be related to genetic differences among individuals. 

### 3.1. Corticosteroids

Corticosteroids are first-line therapy for the management of asthma, both as an inhaled therapy for symptom control and the prevention of exacerbations, and orally and intravenously during acute exacerbations. Corticosteroids are effective in improving asthma symptoms and bronchial hyperresponsiveness through suppression of recruitment and activation of epithelium and inflammatory cells, including but not limited to eosinophils, mast cells, basophils, lymphocytes, and ILC2 cells. However, there is a variable response to inhaled corticosteroid (ICS) therapy in patients with asthma, with some patients not responding despite high doses of therapy, and this variable interindividual response to ICS is highly repeatable, supporting a genetic basis for it [80]. Recent candidate gene studies and genome wide association studies have identified potential phenotypic indicators of response with the hope that clinicians will be able to use this information in guiding patient care and medication use to increase effectiveness and decrease harmful side effects. 

Pharmacogenetic studies of the glucocorticoid pathway have identified genetic polymorphisms on biological candidate genes encoding the glucocorticoid biosynthetic pathway, the receptor heterocomplex and chaperone proteins. These studies identified SNPs in the corticotropin-releasing hormone gene (CRHR1), the heat shock organizing protein gene (STIP1), and glucocorticosteroid receptor gene (NRSC1), which are all associated with increased responsiveness to ICS therapy as measured by increased in FEV1 post ICS treatment [81,82,83,84]. Genotype analysis of nine different SNPs in the CYP3A gene locus on chromosome 7, encompassing the genes CYP3A4, CYP3A5, and CYP3A7, found a significant improvement in asthma control scores, among patients with a CYP3A4*22 variant T-allele [85]. TBX21 encoding for the transcription factor T-bet, which influences naïve T lymphocytes development, was found to have a nonsynonymous variation coding for replacement of histidine 33 with glutamine (H33Q C>G) which is associated with significant improvement in bronchial hyperresponsiveness and asthma control with ICS treatment [86,87]. Both heterozygous and homozygous variants in the FCER2 gene are associated with increased FEV1 in response to treatment with ICS and are indicators of good responders to ICS [88]. Polymorphisms in both vascular endothelial growth factor (VEGFA) and collagen type II alpha 1 chain (COL2A1), a fibrillar collagen found in cartilage, are significantly associated with improved FEV1 response to ICS and [89]. 

In addition to candidate gene studies, GWAS studies have identified a promoter SNP in the glucocorticoid-induced transcript-1 gene (GLCCI1) which is associated with a change in lung function during ICS treatment. Unlike other polymorphisms, mutations in both rs37972 and rs37973 were associated with a smaller improvement of lung function in response to ICS compared to those with the wild-type allele [90]. Another GWAS study identified SNPs in the T gene (rs3127412 and rs6456042) which were associated with a twofold to threefold improvement in FEV1 response to ICS therapy in patients homozygous for the wild-type versus mutant alleles [91]. The T gene has not previously been implicated in asthma or corticosteroid pathology. The T gene is a founding member of an ancient family of genes containing a common protein motif, the T locus [92]. The T locus encodes a product with DNA binding activity which has a role in early vertebrate developmental processes [92]. 

Recent research has highlighted new candidate gene polymorphisms of interest. Polymorphisms in IL-13 rs20541 locus may correlate with therapeutic efficacy; patients carrying the GG allele were more responsive to ICS therapy than those with the GA or AA allele [93]. Vitamin D has multiple effects on the immune system from modulating T cell proliferation causing a switch from a T helper (Th1) phenotype to a Th2 phenotype by inhibiting the synthesis, secretion and release of Th1 cell anti-inflammatory cytokines (IL-4, IL-10) while inducing Th2 cell pro-inflammatory cytokines (IL-1, TNFa, IFN-y) [94]. Reduced vitamin D levels are associated with asthma severity [95] and increased airway hypersensitivity requiring increased doses of ICS [96]. Polymorphisms in the vitamin D receptor gene (VDR) FokI showed an association between patients with the TT genotype and T allele carriers and glucocorticoid-resistance [97]. No associations were found in the VDR Apal [97]. 

The glucocorticoid pathway interacts with other biological pathways which influences the response to ICS monotherapy or when in combination with short-acting β agonists (SABA) or long-acting β agonists (LABA). Adenylyl cyclase type 9 (ADCY9) is an enzyme within the canonical B_2_-adrenergic receptor pathway responsible for producing the second messenger cyclic AMP (cAMP). A SNP in ADCY9 is associated with a strong bronchodilator response to SABA treatment only when used in combination with ICS [98]. Therefore, gene polymorphisms may assist us in understanding interindividual variability in response to ICSs in asthma, and further our understanding regarding steroid resistance in asthma.

### 3.2. β-2 Adrenergic Receptor Pathway

β-2 agonists exert their bronchodilator effects via β-2 adrenoceptors (β_2_Ars) that are heavily expressed on airway smooth muscle (ASM) cells of the lower respiratory tract. Inhaled β agonist binding to the β_2_AR activates a coupling with adenylate cyclase through a trimeric G-protein leading to an increased production of cyclic adenosine monophosphate and protein kinase A resulting in smooth muscle relaxation. Short acting B_2_-agonists (SABA) are used for the treatment of acute symptoms of bronchospasm, while long acting B_2_-agonists (LABA) are used in conjunction with ICS to provide long-term asthma control. 

The most frequently studied gene related response to β-2 agonist treatment response is ADRB2, a small intron-less gene with more than 49 different genetic variants. Early pharmacogenetic studies have demonstrated that patients homozygous for Arg-16 or heterozygous for Arg-16 of the rs1042716 SNP have a greater acute response to SABA bronchodilation compared to Gly-16 [99,100,101,102]. Israel et al. studied the effects of polymorphisms at codon 16 [b2-AR-16] and codon 27 (b2-AR-27) of the β_2_AR in 190 mild asthmatics [103] finding regular SABA use, as opposed to as-needed use, resulted in a decline in peak expiratory flow rate [PEFR] in asthmatics homozygous for ARG-16, while the PEFR in Gly-16 homozygotes remained unchanged. A further study of 78 mild asthmatics enrolled in pairs to match for lung function, found that regular SABA resulted in a significant increase in PEFR Gly-16 homozygotes, while ARG-16 homozygotes had a significant improvement in PEFR during the placebo phase when SABA use was limited, suggesting ARG-16 homozygotes should be treated with other agents such as ipratropium as a reliever, rather than SABA [104]. Early studies of ADRB2 polymorphisms in response to LABA showed that Arg-16 homozygotes experienced a decline in PEFR and a deterioration of symptoms after LABA treatment however two subsequent prospective studies were not able to identify significant differences in PEFR responsiveness to LABA therapy [105,106]. 

Candidate gene studies of β agonist response have also included genes within ADCY9. The Ile-772 Met variant was associated with acute bronchodilation in response to SABA in ICS-treated asthmatics and increased lung function response to LABA and ICS treatment [98,107]. Five SNPs in CRHR2 have been associated with an acute SABA bronchodilator response in three independent cohorts [108]. Rare variants adjacent to ADCY9 and CRHR2 are associated with SABA bronchodilator response in a cohort of patients from Puerto Rico and Mexico [109]. SNPs in the genes that encode the enzymes arginase-1 and arginase-2 metabolize L-arginine (ARG1 and ARG2) within the nitric oxide biosynthetic pathway have been associated with an increased response to acute SABA therapy [110,111]. Additionally, the endothelial nitric oxide synthase gene (NOS3) the variant Asp-298 Glu was associated with increased lung function response to LABA and ICS combination therapy [112]. A polymorphism in the thyroid hormone receptor B-gene (rs892940) was associated with bronchodilator response in a childhood population and two adult populations [113]. 

GWAS performed in non-Hispanic white asthmatics identified a promoter SNP in SPATS2L associated with an acute bronchodilator response to SABA treatment [114]. Another GWAS study showed that SNPs (rs912142) in SPATA13-AS1, an anti-sense RNA that overlaps the gene SPATA13, were associated with increased SABA bronchodilator response [115,116]. Admixture mapping from a GWAS of BDR in a cohort of African Americans with asthma identified SNPs (rs7081864 and rs790336) in the PRKG1 gene which were significantly associated with bronchodilation response to SABA treatment [116,117]. Additionally, admixture mapping identified a novel candidate gene expressed in the lung and bronchial epithelial cells (SLC22A15] with two rare SNPs [rs1281748 and rs1281743] in which individuals carrying the minor allele had enhanced bronchodilation to SABA treatment [109]. 

### 3.3. Leukotrienes

**Figure 3:**
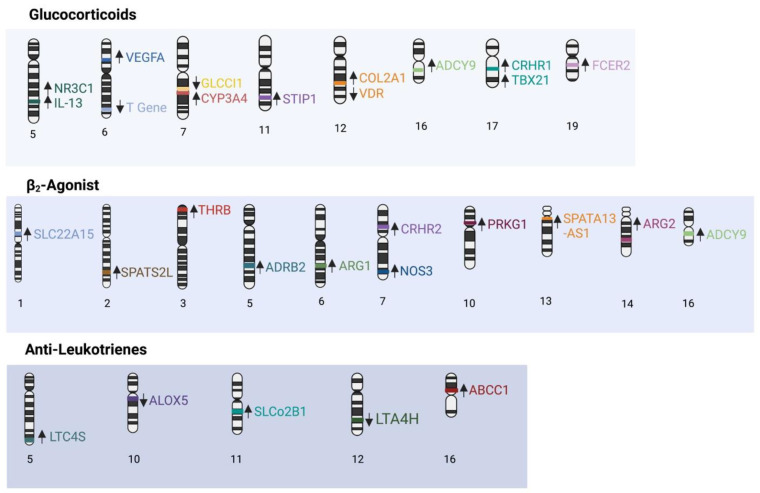
Summary of SNPs associated with treatment response. Arrows indicate improved or worsened response to treatments in the presence of SNPs.

Leukotrienes are a family of lipid mediators with a pivotal and integral role in airway tone and inflammation. There are two types of leukotrienes: LTB_4_ which acts as a potent immune cell chemotactic mediator of inflammation; and the cysteinyl leukotriene group (LTC_4_, LTD_4_, LTE_4_) which are capable of causing significant bronchoconstriction. Leukotriene receptor antagonists, including montelukast and zafirlukast, have anti-inflammatory and anti-bronchoconstrictor properties which improve lung function (FEV1, peak flow) and decrease asthma exacerbations however patients show a wide variety of responsiveness. Variances in the genes coding the enzyme 5-lipoxygenase pathway (coded by ALOX5) showed that wild type or heterozygous ALOX5 promoter and additional SNPs have increased bronchodilator response with montelukast compared to the mutant alleles [118,119]. In contrast the mutant variants in LTC_4_ and MRP1 have been associated with increased lung function response to montelukast and zileuton [120,121]. Additionally, SNPs in gene coding for LTA_4_ showed that patients heterogynous or homozygous for the mutant G allele were at greater risk of having exacerbations while on montelukast [122]. Montelukast is a substrate of organic anion transporting OATP2B1 encoded by the SLCO2B1 gene. A coding variant in SLCO2B1 was found to be associated with increased symptom control in a small cohort of patients [123] however larger cohort studies were not able to replicate these results [124,125]. Table 2 and Figure 3 summarize the association between treatment response and gene polymorphisms. 

## 4. Interactions between Genetic Polymorphisms and Environmental Factors

**Figure 4:**
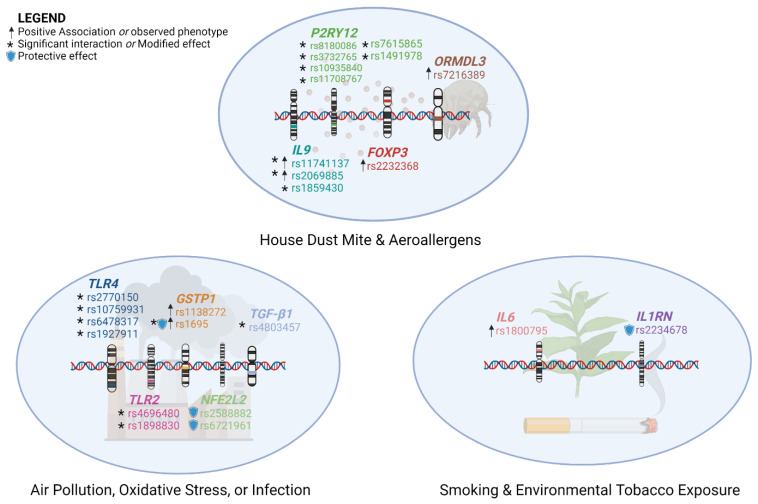
Gene-environmental factor interactions related with modified effects, susceptibility, complications, symptoms, or development of asthma, or asthma exacerbations. SNPs with more than one symbol may demonstrate multiple effects, or may differ depending on genotype, allele pairing, or type of allele (variant vs. wild-type, major vs. minor carrier).

Gene-environment interactions can contribute to the development of asthma when individuals with specific variants of allele genotypes and SNPs are exposed to environmental factors affecting the airway epithelium, such as ambient air pollution, oxidative stress, tobacco smoke, and aeroallergens (Figure 4).

### 4.1. Air Pollution

Gene-environment interactions between amino acid variants of Glutathione S-transferase Pi (GSTP1) and air pollution have been extensively studied in the past. At the Ile-105 locus, Ile-105 homozygotes in areas of high air pollution have a higher risk of asthma compared to those with any Val-105 allele and who are exposed to low air pollution levels [128]. Various studies indicate a val/val genotype at the ile105val locus may be an indicator of injury from oxidative stress in asthmatic children [129], whereas conflicting evidence supports the hypothesis of the val/val genotype acting as a protective genotype [130]. In discussions of gene-environment interactions, children who carry the minor allele for the GSTP1 SNPs rs1138272 or rs1695 (Ile105Val), plus exposure to NO_2_ using land use regression and dispersion modelling have greater susceptibility to injury from air pollution in comparison to major allele carriers, and this susceptibility is highest in children with current asthma, ever asthma, and ever wheeze [131,132]. mRNA expression of Toll-like receptor 2 (TLR2) on human airway epithelial cells has been shown to be involved in the response to air pollution particle [or particulate matter] exposure, whereas Toll-like receptor 4 [TLR4] plays a lesser role through its expression on alveolar macrophages [133]. The rs4696480 and rs1898830 SNPs in the TLR2 gene enhance the effect of exposure of particulate matter in asthmatic children from birth to 8 years of age [134]. Similarly, the SNPs rs2770150 TC, rs10759931 GG, rs6478317 GG, and rs1927911 TT of the TLR4 gene modify the effect of exposure to particulate matter in asthmatics, thus making children susceptible to adverse affects of air pollution when carrying select genotypes of these two genes [134]. Previous studies on TGF-β1 elucidate its role in possible airway inflammation through the release of inflammatory cytokines [135], and in airway remodeling, such as in subepithelial fibrosis [136]. Traffic and tobacco smoke exposure in utero in combination with the TGF-β1 -509TT (rs4803457) genotype increase the risk of asthma [137]. 

### 4.2. Aeroallergens

The combined effect of a genetic predisposition and environmental allergen exposure may influence asthma severity or susceptibility (Figure 3). The *FOXP3* gene is expressed on Tregs cells, and T regulatory 1-like cells (Tr1) cells mediate the Th2 immune response to environmental allergens [138]. The T allele of rs2232368 of the *FOXP3* gene increases the risk of asthma symptoms and onset of atopy to aeroallergens in females [139]. In aeroallergen exposure as well, asthmatics with the *GSTP1* rs1695 SNP carrying the A/A allele are protected from, and those carrying the G allele are more reactive, to household allergens [132]. IL-9 is a cytokine important for immunity and has been shown to be upregulated in asthmatic patients [140]. In asthmatics carrying the dominant genotype for the rs11741137 SNP (T allele) or the rs2069885 SNP (A allele) there is a 4-fold increase in the likelihood of a severe exacerbation with dust mite exposure [141]. 

*ORMDL3* polymorphisms found on chromosome 17q21 have been shown to promote asthma, and when overexpressed, *ORMDL3* increases Th2 cytokine production, and subsequently increases childhood asthma susceptibility [142]. Household allergens such as domestic furred pets, in combination with rs7216389 SNP homozygous carriers of the *ORMDL3* gene, contribute to the significant association of recurrent wheeze in children [143]. 

The P2RY12 (P2Y12 receptor) mediates LTE4-induced pulmonary inflammation in studies when using Chinese hamster ovary cells transfected with human P2Y12 constructs [144]. The rs8180086, rs3732765, rs10935840, and rs11708767 SNPs of P2RY12, can alter airway hyperresponsiveness in asthmatics exposed to a 1.85 µg/g concentration of house dust mite, whereas the rs7615865 and rs149197 SNPs modified bronchodilator reversibility. HDM exposure at 10 µg/g demonstrated similar results, in where the rs3732765, rs10935840, and rs1170876 SNPs demonstrated altered airway hyperresponsiveness, and the rs10935844 and rs8180086 SNPs alter FVC in an asthmatic cohort, thereby providing evidence for a gene-environment interaction between P2RY12 variants and HDM [145].

### 4.3. Oxidative Stress

The *NFE2L2* gene is responsible for encoding a transcription factor is known for its defensive properties against oxidative injury and inflammatory disorders by upregulating antioxidant and detoxifying phase 2 enzymes [146]. NO_2_ exposure can increase the risk of an asthma exacerbation in individuals with respiratory infections [147]. In the *NFE2L2* gene, the rs2588882 and rs6721961 SNPs have a protective effect during infection-induced exacerbation in asthmatics (IIA). These SNPs demonstrate variability in asthmatics depending on their exposure to different polluted areas [148].

### 4.4. Smoking

A gene-smoking environmental interaction exists in asthmatic patients who carry the rs1800795 SNP of the IL-6 gene [149]. Although the uncommon rs2234678 SNP of the IL1RN gene is protective against the development of asthma in those without childhood tobacco exposure, those carrying this SNP with childhood environmental tobacco exposure, are at higher risk of early onset asthma [150]. In a UK cohort, maternal smoking during pregnancy in infants with the rs2234678 GG SNP of IL1RN was also identified as a gene-environment interaction that increased the susceptibility to repeated asthma and persistent asthma in children [151]. Table 3 demonstrates the summary of the effects of gene-environment interactions.

## 5. Conclusions

Asthma is a heterogeneous disease resulting from a complex interplay between genetic and environmental factors. Single nucleotide polymorphisms throughout the genome have diverse impacts on the susceptibility and severity of asthma. While a majority of SNPs affecting asthma lead to increased risk or susceptibility in the carriers, there are specific variants that have a protective effect and decrease the risk when present. The same pattern is also seen in response to drug treatments, where asthmatic patients with specific polymorphisms in their genome demonstrate an improved response to universal asthma medications suggesting that genetic predispositions should be taken into account when prescribing different asthma therapies. 

Studying the genetics of asthma advances our understanding of the pathology, diagnosis, and treatment of this complex disease. Discovering new single nucleotide variations in the genetic material of individuals will not only help identify novel biomarkers and phenotypes but also provide us insights to design beneficial and individualized treatments. 

## Figures and Tables

**Table 1:** Single nucleotide polymorphisms associated with asthma risk and severity.

Gene	Study Design	Race	SNP	Allele	Association	Ref
TSLP	Candidate gene study	Caucasian	rs1837253	C/T	Inversely correlated with risk of asthma	[16]
	Candidate gene study	Middle eastern	rs2289276	C/T	Inversely correlated with the risk of asthma	[17]
			rs2289278	C/G	No association	
	Candidate gene study	Asian	rs3806933	C/T	Associated with asthma susceptibility in adult and childhood asthma	[13]
			rs2289276	C/T		
			rs2289278	C/G	Associated with lung function [FEV1/FVC]	
	Candidate gene study	Caucasian	rs3806933	C/T	Associated with asthma in boys	[14]
			rs2289276	C/T	Associated with higher eosinophil counts in asthmatic girls Associated with lower FEV1 level in asthmatic	
			rs10073816	G/A	No association	
			rs11466749	A/G	The presence of allergic rhinitis in asthmatic children strengthened the association of the rs11466749 genotype with asthma	
	Candidate gene study	Mixed	rs1837253	C/T	Associated with a reduced risk of asthma in males	[15]
			rs2289276	C/T	Associated with a reduced risk of asthma in females	
IL-33	Candidate gene study	Asian	rs4742170	C/T	Associated with risk of higher FeNO at baseline	[22]
			rs2381416	A/C	No association	
			rs928413	A/G	No association	
			rs992969	A/G	No association	
	GWAS	Caucasian	rs146597587	G/C	Associated with lower eosinophil count and reduced risk of asthma	[25]
	Candidate gene study	Caucasian	rs1412420	C/A	Associated with adult-onset asthma	[23]
			rs7037276	G/C		
			rs996029	A/T		
	Candidate gene study	Middle eastern	rs1342326	A/C	Associated with higher risk of asthma	[24]
			rs3939286	G/A		
IL1RL1	Candidate gene study	Caucasian	rs11685480	G/A	Associated with lower blood eosinophil count	[30]
			rs1420102	C/A		
			rs1041973	C/A	Associated with decreased risk of developing asthma	
	Candidate gene study	Puerto Rican	rs1921622	A/G	No association	[29]
	Candidate gene study	Asian	rs1420102	C/A	Associated with higher risk of asthma	[28]
			rs13431828	C/T	No association	
	Candidate gene study	Mixed	rs13431828	T/C	Associated with increased risk of exacerbation	[32]
			rs1041973	A/C	No association	
			rs1946131	G/A		
			rs1420101	G/A	Associated with lower serum sST2 levels	
			rs1921622	G/A		
			rs10204137	G/A		
IL-13	Candidate gene study	Mixed	rs1295686	C/T	Associated with increased risk of asthma	[37]
	Candidate gene study	Middle eastern	rs20541	G/A	Associated with increased risk of asthma	[41]
	Candidate gene study	Middle eastern	rs1295686	C/T	Associated with increased risk of asthma	[35]
			rs20541	G/A		
			rs1800925	G/A	No association	
			rs762534	C/A		
	Candidate gene study	Asian	rs20541	G/A	Associated with AHR	[39]
IL-4	Candidate gene study	Caucasian	rs2243250	C/T	Associated with higher serum IgE levels	[74]
	Candidate gene study	Asian	rs2227284	T/G	Associated with reduced risk of asthma	[47]
			rs2243250	T/C	No association	
			rs2070874	T/C		
			rs2243290	A/C		
		Middle eastern	rs2243250	T/C	Associated with asthma and allergic rhinitis	[48]
			rs2227284	T/G		
			rs2070874	T/C	No association	
HLA	GWAS	Mixed	rs9272346	A/G	Associated with increased risk asthma	[51]
ADAM33	Candidate gene study	Middle eastern	rs2280091	A/G	No association	[59]
			rs3918396	G/A		
			rs2280089	G/A		
			rs511898	C/A		
	Candidate gene study	Asian	rs44707	G/C	Associated with severe asthma	[61]
			rs2280089	G/A	Associated with increased risk asthma	
			rs2787094	G/C	Associated with less severe asthma	
			rs612709	G/A	No association	
			rs511898	C/A		
			rs2280091	A/G		
			rs528557	G/C		
			rs3918396	G/A		
	Candidate gene study	South Asian	rs528557	G/C	Associated with increased risk asthma	[63]
			rs597980	C/T		
			rs511898	C/A		
			rs44707	G/C		
			rs2787094	G/C		
	Candidate gene study	Asian	rs528557	G/C	Associated with low severity asthma	[62]
			rs598418	C/T	Associated with high severity asthma	
			rs44707	G/C	No association	
			rs2853209	A/T		
			rs597980	C/T		
			rs11905233	G/A		
			rs2787094 rs3746631	G/C		
	Candidate gene study	Caucasian	rs2787094	G/C	Associated with mild asthma	[75]
	Candidate gene study	Caucasian	rs511898	G/A	No association	[60]
			rs3918395	G/T		
			rs3918396	G/A		
			rs528557	G/C		
			rs44707	G/C		
			rs597980	C/T		
			rs574174	G/A		
			rs2280091	A/G		
			rs2280090	C/T		
			rs2787094	G/C		
	Candidate gene study	Caucasian	rs44707	G/C	Associated with increased risk of asthma	[64]
			rs2787095	G/T	No association	
			rs2485700	G/A		
			rs2280091	A/G		
	Candidate gene study	Asian	rs678881	G/C	Associated with increased risk of asthma	[65]
			rs2280089	G/A	No association	
			rs2853209	A/T		
VDR	Candidate gene study	Caucasian	rs731236	C/T	Associated with increased risk of asthma	[69]
			rs7975232	A/C	Associated with reduced mRNA gene expression in asthmatic group.	
	Candidate gene study	North African	rs731236	C/T	Associated with increased risk of asthma	[72]
			rs7975232	A/C		
			rs2228570	T/C		
			rs1544410	G/A		
	Candidate gene study	North African	rs7975232	A/C	No association	[70]
			rs731236	C/T	Associated with increased risk of asthma	
			rs1544410	G/A		
	Candidate gene study	Caucasian	rs731236	C/T	Associated with increased risk of asthma	[71]
			rs7975232	A/C		
	Candidate gene study	Caucasian	rs731236	C/T	Associated with increased risk of asthma	[76]
			rs7975232	A/C	No association	
			rs1544410	G/A		
	Candidate gene study	Asian	rs1544410	G/A	Associated with increased risk of asthma	[77]
			rs7975232	A/C		
			rs2228570	T/C		

Definition of abbreviations: AHR = airway hyperresponsiveness; FeNO: fractionatal exhaled nitric oxide; FEV1 = forced expiratory volume in one second; FVC = forced vital capacity; GWAS = genome-wide association studies; IgE = immunoglobulin-E; IL- = interleukin-; mRNA = messenger ribonucleic acid; SNP = single nucleotide polymorphism; TSLP = thymic stromal lymphopoietin; VDR = vitamin D receptor.

**Table 2:** Single Nucleotide polymorphisms associated with treatment response.

Gene	Study Design	Race	SNP	Allele	Drug Class	Response Phenotype	Ref
CRHR1	Candidate gene study	Mixed	rs242941	C/A	ICS	Variants improves FEV1	[81]
			rs1876828	C/T			
STIP1	Candidate gene study	Asian/Caucasian	rs2236647	C/T	ICS	Variants improves FEV1	[82,83]
			rs6591838	A/G			
			rs1011219	G/A			
			rs4980524	A/C			
NR3C1	Candidate gene study	Caucasian	rs41423247	G/C	ICS	Variants improve FEV1	[84]
TBX21	Candidate gene study	Mixed	rs2240017	C/G	ICS	Variant improved airway hyperresponsiveness	[86,87]
GLCCI1	GWAS	Mixed	rs37973	G/T	ICS	rs37973 Wild-type allele had improved FEV1 and response to ICS compared to mutant allele	[90]
			rs37972	A/T			
T gene	GWAS	Mixed	rs3127412	A/C	ICS	Wild-type allele had improved FEV1 and response to ICS compared to mutant allele	[91]
			rs6456042	C/A			
			rs3099266	A/T			
ADCY9	Candidate gene study	Mixed	rs2230739	T/C	ICS B2 agonist	Variants improved bronchodilator response	[98]
CYP3A4	Candidate gene study	Mixed	CYP3A4*22 allele		ICS	T-allele variants improved asthma control	[85]
FCER2	Candidate gene study	Mixed	rs28364072	A/G	ICS	Variants improve FEV1	[88,126,127]
VEGFA	Candidate gene study	Mixed	rs3025039	C/T	ICS	Variants improve FEV1	[89]
COL2A1	Candidate gene study	Mixed	rs3809324	G/T	ICS	Variants improve FEV1	[89]
IL-13	Candidate gene study	Mixed	rs20541	C/A	ICS	Variants improved FEV1	[93]
Vitamin D receptor	Candidate gene study	Middle eastern	FokI rs228570 Apal Rs7975232	T/C A/C	ICS	FokI variants are associated with GC-resistance	[97]
ADRB2	Candidate Gene Study	Mixed	rs1042716	G/T	SABA	Short-term use: Arg 16 has greater bronchodilation response than Gly 16 Long-term use: no allele effect	[99,100,101,102]
			rs11959427	A/C			
ADCY9	Candidate gene study	Mixed	rs2230739	T/C	SABA	Acute FEV1 bronchodilation	[98]
CRHR2	Candidate Gene Study	Mixed /South American	rs7793837	A/T	SABA	Acute FEV1 bronchodilation	[108,109]
ARGI	Candidate Gene Study	Caucasian	rs2781659	A/G	SABA	Acute FEV1 bronchodilation	[110,111]
			rs2781667	C/T			
ARG2	Candidate Gene Study	Caucasian	rs7140310	T/G	SABA	Acute FEV1 bronchodilation	[111]
			rs10483801	C/A			
THRB	Candidate Gene Study	Caucasian	rs892940	G/A	SABA	Acute FEV1 bronchodilation	[113]
SPATS2L	GWAS	Caucasian	rs295137	C/T	SABA	Acute FEV1 bronchodilation	[114]
SPATA13-AS1	GWAS	Mixed/African American	rs912142	A/G	SABA	Acute FEV1 bronchodilation	[115,116]
SLC22A15	Admixture mapping	South American	rs1281748	A/G	SABA	Acute FEV1 bronchodilation	[109]
			rs1281743	G/A			
PRKG1	Candidate Gene Study	Mixed	rs7081864 rs7903366	Admixture mapping	SABA	Acute FEV1 bronchodilation	[116,117]
ADRB2	Candidate Gene Study	Mixed	rs1042716	G/T	LABA	No allele effect	[105,106]
			rs1800888	C/T			
ADCY9	Candidate Gene Study	Asian	rs2230739	T/C	LABA	Long-term increased FEV1 response	[107]
NOS3	Candidate Gene Study	Mixed	rs1799983	A/T	LABA	Acute FEV1 bronchodilation in Asp 298 Glu	[112]
ALOX5	Candidate Gene Study	Mixed	rs892690	C/T	Antileukotriene	Wild type allele increased BDR	[118,119]
			rs2115819	G/A			
			rs10507391	C/A			
			rs4986832	G/A			
			rs4987105	C/T			
LTC_4_	Candidate Gene Study	Mixed	rs272431	G/T	Antileukotriene	Mutant allele increased BDR	[120,121]
			rs730012	A/C			
MRP1	Candidate Gene Study	Mixed	rs119774	C/T	Antileukotriene	Mutant allele increased BDR	[121]
			rs215066	G/A			
LTA4	Candidate Gene Study	Mixed	rs2660845	A/G	Antileukotriene	Wild type allele decreased asthma exacerbations	[122]
SLCo2B1	Candidate Gene Study	Mixed	rs12422149	G/A	Antileukotriene	Increased symptom control	[123]

Definition of abbreviations: FEV1 = forced expiratory volume in one second; GC = glucocorticoid; ICS = inhaled corticosteroid; LABA = long-acting β-agonist; SABA = short-acting β-agonist; SNP = single nucleotide polymorphism.

**Table 3:** Combined effects of gene-environmental interactions.

Gene	Study Design	Race	SNP	Allele	Association	Ref
FOXP3	Candidate gene	Brazilian	rs2232368	C/T	Associated with asthma susceptibility and onset of atopy in females.	[139]
TGF-B1	Candidate gene	Mixed	rs4803457	C/T	-509TT genotype carriers have higher susceptibility to early-onset childhood asthma	[137]
GSTP1	Candidate gene	Canadian, Swedish, German, Netherlands (six birth cohorts)	rs1138272	C/T	Minor allele carriers with NO_2_ exposure have higher risk to current asthma, ever asthma, and injury from air pollution compared to major allele carriers	[131]
			rs1695	A/G*	Minor allele carriers for both rs1138272 and rs1695 exposed to traffic and NO_2_ may be at an increased risk of asthma, current asthma, and ever asthma. *A/A genotype has protective properties against complications from household allergens, G allele makes asthmatics more prone to complications	[131,132]
NFE2L2	Candidate gene	Hungarian	rs2588882	T/G	Protective effect during infection-induced exacerbation in asthmatics [IIA]	[148]
			rs6721961	G/T		
TLR2	Candidate gene	Netherlands	rs4696480	T/A	Modifies effect of exposure of air pollution on asthma from birth to 8 years of age	[134]
			rs1898830	A/G		
TLR4	Candidate gene	Netherlands	rs2770150	A/G	Modify the effect of exposure to air pollution on asthma	[134]
			rs10759931	G/A		
			rs6478317	A/G		
			rs1927911	C/A		
ORMDL3	Candidate gene	Danish, delivered in Copenhagen	rs7216389	T/C	Significant association with risk of recurrent wheeze in homozygous variant allele carriers	[143]
IL9	GWAS	Mixed	rs11741137	C/T	Significant interactions of the rs11741137, rs2069885, and rs1859430 SNPs with dust mite allergen exposure. Increase in the likelihood of exacerbation for dominant genotype carriers [rs11741137, rs2069885] and with increased dust mite exposure	[141]
			rs2069885	G/A		
			rs1859430	G/A		
P2RY12	Candidate gene	Mixed	rs8180086	G/A	Modified airway hyperresponsiveness values with HDM exposure	[145]
			rs3732765	G/A		
			rs10935840	A/G		
			rs11708767	G/A		
			rs7615865	A/T	Modified bronchodilator reversibility with HDM exposure	
			rs1491978	G/C		
IL6		Chinese	rs1800795	G/C	Gene-smoking environmental interaction increases risk of asthma	[149]
IL1RN	Candidate gene	Mixed [150] or Caucasian [151]	rs2234678	A/G	Protective properties in the development of asthma in those without childhood environmental tobacco exposure; those with childhood exposure, were at higher risk of early onset asthma. Maternal smoking during pregnancy in infants with the GG genotype had increased susceptibility to repeated asthma and persistent asthma in children	[150,151]

## Data Availability

Not applicable.
